# Multiple forms of vitamin B_6_ regulate salt tolerance by balancing ROS and abscisic acid levels in maize root

**DOI:** 10.1007/s44154-022-00061-2

**Published:** 2022-09-19

**Authors:** Chongchong Lu, Yuan Tian, Xuanxuan Hou, Xin Hou, Zichang Jia, Min Li, Mingxia Hao, Yanke Jiang, Qingbin Wang, Qiong Pu, Ziyi Yin, Yang Li, Baoyou Liu, Xiaojing Kang, Guangyi Zhang, Xinhua Ding, Yinggao Liu

**Affiliations:** 1 State Key Laboratory of Crop Biology, Shandong Provincial Key Laboratory for Biology of Vegetable Diseases and Insect Pests, College of Plant Protection; Shandong Agricultural University, Taian, 271018 Shandong China; 2 Shandong Pengbo Biotechnology Co., LTD, Taian, 271018 China; 3 Shandong Agriculture and Engineering University, Jinan, 250000 Shandong China; 4 Yantai Academy of Agricultural Sciences, Yantai, 265500 Shandong China; 5 Shandong Xinyuan Seed Industry Co., LTD, Taian, 271000 China

**Keywords:** Vitamin B_6_, Abscisic acid, Reactive oxygen species, Salt stress, *smk2*

## Abstract

Salt stress causes osmotic stress, ion toxicity and oxidative stress, inducing the accumulation of abscisic acid (ABA) and excessive reactive oxygen species (ROS) production, which further damage cell structure and inhibit the development of roots in plants. Previous study showed that vitamin B_6_ (VB_6_) plays a role in plant responses to salt stress, however, the regulatory relationship between ROS, VB_6_ and ABA under salt stress remains unclear yet in plants. In our study, we found that salt stress-induced ABA accumulation requires ROS production, in addition, salt stress also promoted VB_6_ (including pyridoxamine (PM), pyridoxal (PL), pyridoxine (PN), and pyridoxal 5′-phosphate (PLP)) accumulation, which involved in ROS scavenging and ABA biosynthesis. Furthermore, VB_6_-deficient maize mutant *small kernel2* (*smk2*) heterozygous is more susceptible to salt stress, and which failed to scavenge excessive ROS effectively or induce ABA accumulation in maize root under salt stress, interestingly, which can be restored by exogenous PN and PLP, respectively. According to these results, we proposed that PN and PLP play an essential role in balancing ROS and ABA levels under salt stress, respectively, it laid a foundation for VB_6_ to be better applied in crop salt resistance than ABA.

**Supplementary Information:**

The online version contains supplementary material available at 10.1007/s44154-022-00061-2.

## Introduction

Soil salinity is one of the most inclusive abiotic stresses, limits crop growth and production (Asgher et al., 2017) and causes osmotic stress, which is followed by ion poisoning and oxidative stress (Bagri et al., 2018; Hao et al., 2021; Hernandez et al., 2001; Mittler, 2017). Osmotic stress affects the accumulation of ABA, which leads to the closure of stomata and greatly reduces photosynthesis, ultimately leading to plant growth inhibition. ABA is the key plant phytohormone in resisting the harsh conditions caused by abiotic stress (Banerjee and Roychoudhury, 2017; Mehrotra et al., 2014; Vishwakarma et al., 2017). Abiotic stress, such as drought stress, salt stress and heat stress, strongly induces ABA biosynthesis in rice and maize (Dinler et al., 2014; Huang et al., 2018; Lu et al., 2019). Moreover, ABA produces a defense response by inducing the accumulation of ROS in plant cells (Sakamoto et al., 2008), which further causes deleterious effects on plant cells. In brief, although ABA and ROS regulate each other under abiotic stress, the mechanism is still unclear. Abiotic stress triggers the expression of a series of ABA biosynthesis genes, including zeaxanthin oxidase (ZEP), molybdenum cofactor sulfurase (MCSU) and ABA-aldehyde oxidase (AAO) (Vishwakarma et al., 2017). The first step of ABA biosynthesis is initiated by ZEP in *Nicotiana plumbaginifolia* (Marin et al., 1996), which is involved in stress regulation by inducing ABA accumulation in seed germination (Xiong et al., 2001; Xiong and Zhu, 2003). Viviparous14 (Vp14), which has the same function as 9-cis-epoxycarotenoid dioxygenase (NCED) in maize, was first cloned in 1996 by Tan et al. (Schwartz et al., 2003; Tan et al., 1997). AAO3 is responsible for the final step of the ABA biosynthesis process with the help of a molybdenum (Mo) cofactor, oxidizing the abscisic aldehyde to abscisic acid (Vishwakarma et al., 2017). Previous results indicated that pyridoxal phosphate (PLP) coenzyme was essential for the activity of molybdenum cofactor sulfurase (Heidenreich et al., 2005). The concept of oxidative stress, which is a biochemical process caused by the imbalance between the production of antioxidants and oxidants, which further induces cell damage and changing cellular physiology (Rosado-Perez et al., 2018), was formulated in 1985 (Sies, 2015; Sies and Cadenas, 1985).

ROS is a general term for a group of substances with strong oxidation in plants, mainly including singlet oxygen (^1^O_2_), hydroxyl radical (OH^−^), superoxide anion (O_2_^−^) and hydrogen peroxide (H_2_O_2_) (Ahanger et al., 2017; Waszczak et al., 2018). Over the past decade, ROS were thought to regulate various biological processes, and was induced by virous environmental stresses (Jimenez-Quesada et al., 2016; Leng et al., 2017; Saini et al., 2018; Tognetti et al., 2017; Xie et al., 2014; You et al., 2014). Salt stress-induced ROS are secondary damage signal that ultimately affect the membrane structure, root development and hormone metabolism inside and outside the plants cell (Ahmad et al., 2008; Bienert et al., 2006; Schmidt et al., 2013; Yang et al., 2007; You and Chan, 2015). Previous studies have shown that ROS disrupt auxin transport, thereby affecting the gravitropism of primary roots (PRs) (Joo et al., 2001; Joo et al., 2005). ROS also regulate cell proliferation and differentiation in the root (Tsukagoshi et al., 2010). Furthermore, in vivo ROS homeostasis plays a central role in cell division, differentiation and regulating lateral root (LR) emergence (Biswas et al., 2019; Orman-Ligeza et al., 2016; Xu et al., 2018). However, excessive ROS accumulation damages plant cellular structure and negatively modulates the development of lateral root emergence under high salt stress (Ahanger et al., 2017). In addition, exogenous hydrogen peroxide treatment inhibited primary root (PR) elongation and the emergence of lateral roots (LRs) (Orman-Ligeza et al., 2016), which also indicated that excessive ROS negatively regulated root development. ROS metabolism also contributes to root growth under drought stress (Dalal et al., 2018). In summary, these studies show that ROS are a double-edged sword for plant development, and maintaining the dynamic balance of ROS in plants is crucial for root development.

Vitamin B_6_ (VB_6_) contains pyridoxamine (PM), pyridoxal (PL), pyridoxine (PN), and their 5′-phosphorylated forms (Drewke and Leistner, 2001; Yang et al., 2017), which were identified as singlet oxygen antioxidants (Bilski et al., 2000). In addition, pyridoxal 5′-phosphate (PLP) plays an essential role in a variety of enzyme systems as a cofactor (Rosenberg, 2012). Previous results showed that oxidative stress strongly induced the expression of pyridoxine biosynthesis 1.2 (PDX1.2), which participates in VB_6_ biosynthesis in Arabidopsis (Moccand et al., 2014). More interestingly, overexpression of the *PDX-II* gene also enhanced potato tolerance to salt stress (Bagri et al., 2018). In addition, VB_6_ biosynthesis-deficient mutant plants are more sensitive to salt stress (Gonzalez et al., 2007; Titiz et al., 2006). In maize, the VB_6_ biosynthesis-deficient plants *small kernel2* (*smk2*) homozygous mutants exhibit an embryonic lethal phenotype, and *SMK2* has the similar function with Arabidopsis VB_6_ biosynthesis gene *PDX2.1* (Yang et al., 2017). However, the mechanism of *SMK2* in regulating salt-resistant still remains unclear.

Root system is an extremely important nutrient absorption organ for plants and sensitive to environmental change (Kolb et al., 2017; Shahzad and Amtmann, 2017; Su et al., 2017; Sun et al., 2017). The root system contains PRs, LRs and adventitious roots (Olatunji et al., 2017). LRs are more sensitive than PR in responding to salt stress, and the growth and development of LRs are regulated by many plant hormones, including ethylene, auxin, abscisic acid and cytokinin (Ilina et al., 2018; Jing and Strader, 2019; Lu et al., 2019; Qin and Huang, 2018). Under salt stress, ABA severely inhibited the development of maize LRs (Lu et al., 2019). Based on the abovementioned studies, ROS, ABA and VB_6_ play essential roles in plant root development under salt stress, however, the relationship among them remained unclear yet. Here, we further explored the role of VB_6_ in balancing salt stress-induced ROS and ABA content in maize roots.

In our study, we found that salt stress simultaneously induced ROS production, ABA and VB_6_ (including PM, PL, PN, PLP) accumulation in maize roots. Furthermore, PN exogenous can eliminate salt stress-induced ROS accumulation and enhance root resistance to salt stress. In addition, VB_6_-deficient *smk2* heterozygous plants were more susceptible to salt stress and failed to scavenge excessive ROS and induce ABA accumulation under salt stress. Further study showed that exogenous PN rescued the salt stress-susceptible phenotype of heterozygous *smk2*, on the other hand, exogenous PLP restored salt stress-induced ABA accumulation in heterozygous *smk2* by acting as a coenzyme to promote AAO activity, which is involved in ABA biosynthesis in maize roots. In summary, PN and PLP act as antioxidants and coenzymes, respectively, which can eliminate excessive ROS and regulate the biosynthesis of ABA under salt stress, so as to balance the accumulation of ROS and ABA and make maize roots better adapt to salt stress.

## Results

### ROS production is required for ABA accumulation induced under salt stress

**Fig. 1:**
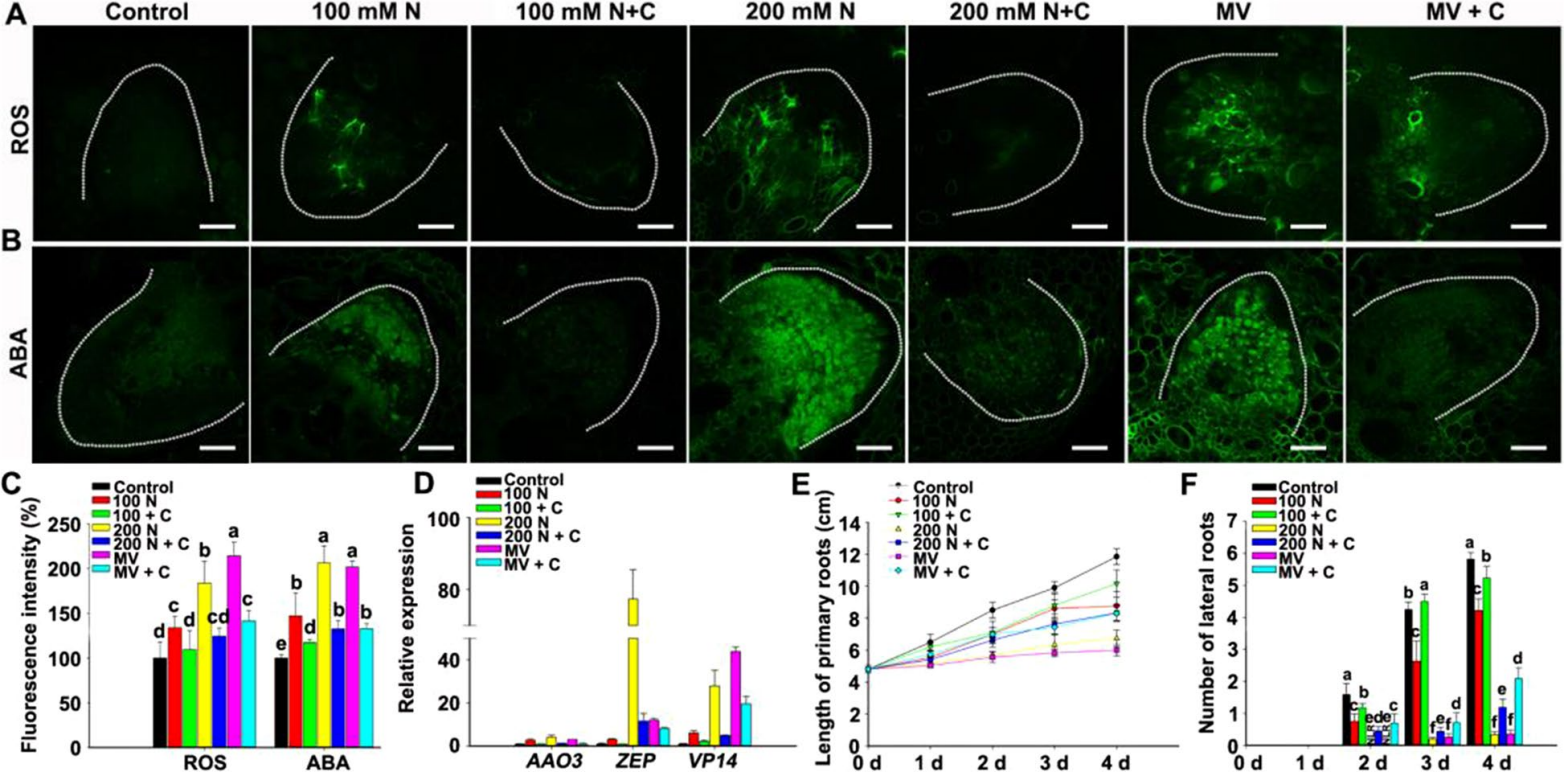
ROS regulate the accumulation of ABA in lateral roots induced by salt stress. **A**, **B** ROS and ABA fluorescence in LRs of 6-d-old seedlings subjected to H_2_O (Control), 100 mM NaCl (N), 100 mM NaCl + 100 μM CAT (Catalase), 200 mM NaCl, 200 mM NaCl + 100 μM CAT, 10 μM MV (Methyl viologen) and 10 μM MV + 100 μM CAT for 24 h. ROS and ABA fluorescence were detected by using the fluorescent dye H_2_DCFDA and immunofluorescence in LRs, respectively. Bar = 50 μm. The white thread represents the profile of the LR. **C** ROS and ABA fluorescence intensity of A and B. **D** qRT–PCR analysis of AAO3, ZEP and VP14 expression in maize roots of 6-d-old seedlings subjected to H2O (Control), 100 mM NaCl (N), 100 mM NaCl + catalase (CAT, C), 200 mM NaCl, 200 mM NaCl + CAT, 10 μM MV (Methyl viologen) and 10 μM MV + CAT for 24 h. **E** Length of PRs after different treatments for 4 d. PRs is from independent maize seedling (*n* ≥ 6). **F** Number of LRs (*n* ≥ 6) after different treatments for 4 d. LRs is from independent maize seedling (*n* ≥ 6). Image J software was used for quantifying fluorescence intensity. The different letters represent significant differences (*P* < 0.05, based on one-way ANOVA)

Many studies have shown that ROS are involved in high salinity-inhibited root growth (Golldack et al., 2014; Miller et al., 2010). Our previous work revealed that ABA inhibited LR emergence by affecting the polar location of PIN1 in maize (Lu et al., 2019). To further reveal the regulatory relationship between ROS and ABA under salt stress, the ROS inducer methyl viologen (MV), which inhibits electron transfer in the electron transfer chain and leads to ROS production (Li et al., 2017), was used to mimic oxidative stress. We observed that the elongation of PRs and the number of LRs were strongly inhibited with the increase of MV, and we choose 10 μM MV for the following experiments (Fig. S1). Our results showed that ROS marker fluorescence signal was stronger than that of control under NaCl or MV treatment (Fig. 1A). And the green immunofluorescence of ABA showed significant enhancement after NaCl and MV treatment (Fig. 1B). Similarly, quantitative analysis of the fluorescence intensity of ROS clearly showed that the fluorescence intensity under 100, 200 mM NaCl and 100 μM MV treatments was 27%, 68% and 115% higher than that of the control, respectively (Fig. 1C), and the ABA immunofluorescence intensity under 100, 200 mM NaCl and 100 μM MV treatment increased by 50%, 110% and 108% (Fig. 1C), respectively. In addition, the ABA biosynthesis genes (*AAO3*, *ZEP* and *VP14*) were significantly upregulated after NaCl and MV treatment (Fig. 1D). Furthermore, NaCl and MV treatment significantly inhibited PR elongation and strongly decreased the number of LRs (Fig. 1E and F). Therefore, exogenous catalase (CAT), a ROS scavenger, partially recovered PR elongation and the number of LRs under salt stress (Fig. 1E and F). And CAT significantly decreased the ROS marker fluorescence intensity (Fig. 1A and C), the ABA immunofluorescence intensity and the expression of ABA biosynthesis genes in maize roots (Fig. 1B-D). These results suggested that ROS play an essential role for ABA accumulation and the inhibition of root growth under salt/oxidative stress.

### ROS is involved in NaCl induced-VB_6_ accumulation

**Fig. 2:**
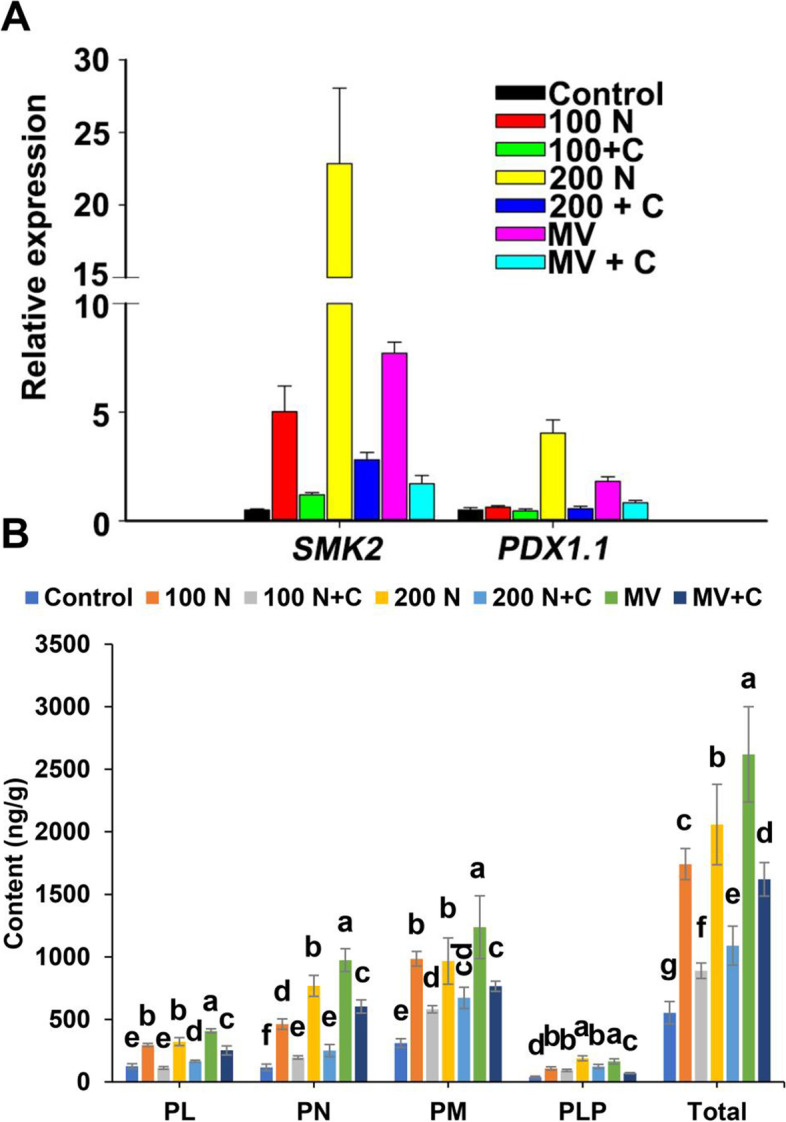
Salt and oxidative stress induced VB_6_ accumulation in maize roots. **A** qRT–PCR analysis of *SMK2* and *PDX1.1* expression in maize roots of 6-d-old seedlings subjected to H_2_O (Control), 100 mM NaCl (N), 100 mM NaCl + CAT, 200 mM NaCl, 200 mM NaCl + CAT, 10 μM MV (Methyl viologen) and 10 μM MV + CAT for 24 h. **B** HPLC detection of the contents of PL, PN, PM, and PLP after different treatments for 24 h. The different letters represent significant differences (*P* < 0.05, based on one-way ANOVA)

Previous study also showed that VB_6_ biosynthesis genes were important for oxidative stress and salt stress responses in plants (Bagri et al., 2018; Titiz et al., 2006). Thus, we hypothesize that VB_6_ is also induced by salt stress. To prove our hypothesis, we detected the expression of VB_6_ biosynthesis genes (*SMK2* and *PDX1.1*) and VB_6_ content under salt stress in maize root. The expression of *SMK2* and *PDX1.1* was dramatically upregulated, especially the expression of *SMK2,* which increased 8-, 5- and 22-fold compared with that of the control under 10 μM MV, 100 and 200 mM NaCl treatment (Fig. 2A), respectively. As expected, exogenous CAT also restored the expression of the *SMK2* and *PDX1.1* induced by NaCl (Fig. 2A), and 10 μM exogenous MV also induced the expression of *SMK2* and *PDX1.1*. We also measured the content of VB_6_, including PL, PN, PM and PLP, by using high-performance liquid chromatography (HPLC) in maize roots. NaCl treatment significantly increased the four VB_6_ isoforms accumulation in maize root (Fig. 2B), which was marked decreased by exogenous CAT (Fig. 2B). Similarly, exogenous CAT partially restored the expression of ABA biosynthesis genes (*AAO3*, *ZEP*, *VP14*) and VB_6_ biosynthesis genes (*SMK2* and *PDX1.1*) and the content of different components of VB_6_ induced by MV (Fig. 1D, 2A and B). These results suggest ROS production is essential for salt/oxidative stress-induced VB_6_ accumulation.

### Exogenous PN decreased ROS and ABA accumulation

**Fig. 3:**
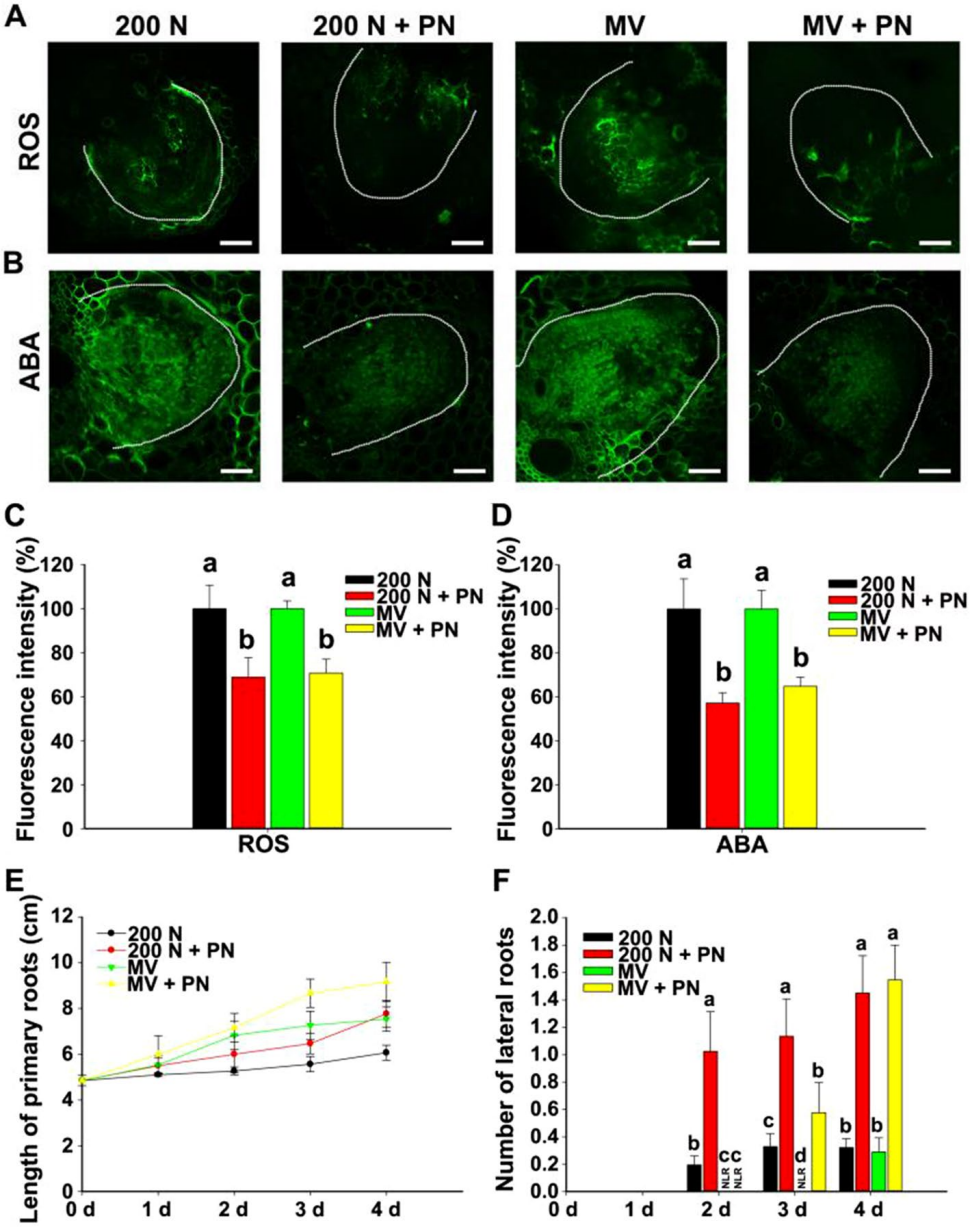
Exogenous PN reduced ROS and ABA accumulation under salt stress. **A**, **B** ROS and ABA fluorescence in LRs of 6-d-old seedlings subjected to 200 mM NaCl (N), 200 mM NaCl + 100 μM PN, 10 μM MV (methyl viologen) and 10 μM MV + 100 μM PN for 24 h. ROS and ABA fluorescence were detected by using the fluorescent dye H_2_DCFDA and immunofluorescence in LRs, respectively. Bar = 50 μm. The white thread represents the profile of the LR. **C** ABA fluorescence intensity of (**A**). **D** ROS fluorescence intensity of (**B**). Image J software was used for quantifying fluorescence intensity. The different letters represent significant differences (*P* < 0.05, based on one-way ANOVA). **E** Length of PRs after different treatments for 4 d. PRs is from independent maize seedling (n ≥ 6). **F** Number of LRs after different treatments for 4 d. LRs is from independent maize seedling (n ≥ 6). Different letters represent significant differences (*P* < 0.05, based on one-way ANOVA)

VB_6_ and its derivatives were identified as antioxidants by quenching singlet oxygen, especially PN, which has a strong singlet oxygen scavenging capacity (Bilski et al., 2000). To elucidate the function of different components of VB_6_, we performed the following experiments. Firstly, we compared the ability of PN, PM PL and PLP in scavenging ROS. To test the ability of PN to scavenge ROS, PN was applied under NaCl and MV treatment. Supplementation with exogenous PN dramatically decreased the ROS and ABA fluorescence intensity and brightness under NaCl or MV treatment (Fig. 3A-D, Fig. S2), respectively. Furthermore, PN also stronger rescued the inhibition of LRs and PRs development by NaCl (200 mM) than PL and PM, while PLP failed to restore the growth of LR and PR under the 200 mM NaCl treatment (Fig. S3), similarly, PLP also failed scavenging ROS accumulation (Fig. S2). Similarly, exogenous PN supplementation also promoted PR elongation and increased the number of LRs under the NaCl and MV treatment (Fig. 3E and F), indicating that PN effectively scavenge ROS accumulation induced by NaCl. In addition, PN also significantly decreased the expression of ABA biosynthesis genes (*AAO3, ZEP* and *VP14*) induced by NaCl and MV (Fig. S4). These results indicate that PN may be involved in scavenging excessive ROS and ABA biosynthesis under salt treatment.

### *smk2* heterozygous plants were more sensitive to salt stress

**Fig. 4:**
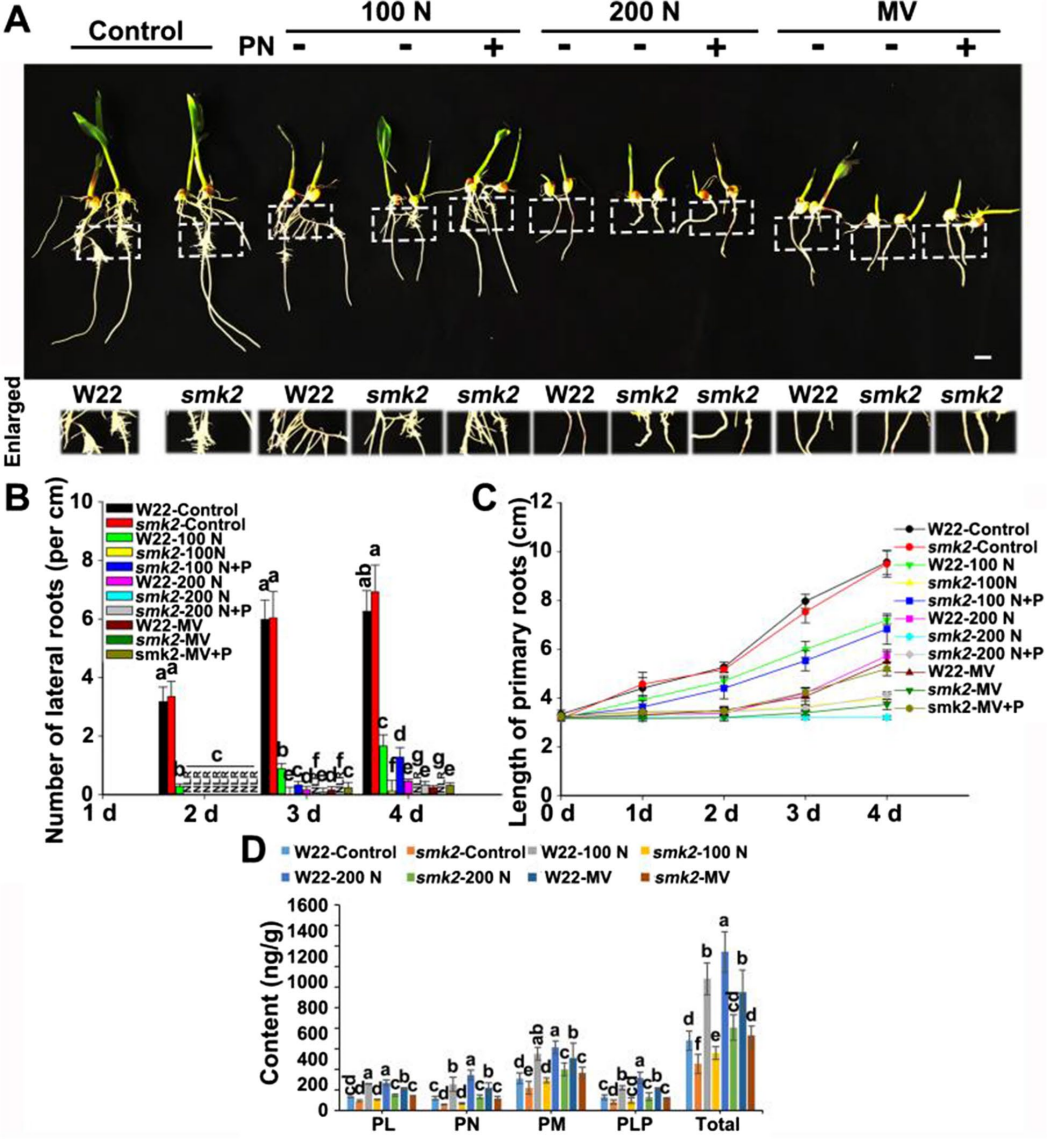
*smk2* heterozygous plants were more sensitive than the W22 plants to salt stress. **A** Phenotypes of W22 and *smk2* heterozygous suffered from H_2_O (Control), 100 mM NaCl (N), 200 mM NaCl and 10 μM MV (methyl viologen), − and + means with or without 100 μM PN. Bar = 1 cm. The white dotted lines show the enlarged part. **B**  LRs number of (A) after different treatments for 4 d. LRs are from independent maize seedling (*n* ≥ 6).  **C** The PRs length of (A) after different treatments for 4 d. PRs are from independent maize seedling (*n* ≥ 6). **D** HPLC detection of the contents of PL, PN, PM, and PLP after different treatments for 24 h in W22 and *smk2* heterozygous plants. The different letters represent significant differences (*P* < 0.05, based on one-way ANOVA)

Next, *smk2* heterozygous plants was identified and used to further reveal the effect of endogenous VB_6_ on salt resistance and antioxidation in maize (Fig. S5). Our results showed that the heterozygous *smk2* phenotype exhibited a similar phenotype to that of the wild type (W22) under normal conditions (Fig. 4A). However, the developmental state of heterozygous *smk2* was weaker than that of the control under salt stress (Fig. 4A). More seriously, PRs length and the density of lateral roots in heterozygous *smk2* exhibited an extensive reduction compared with that of the W22 under salt stress (Fig. 4A-C). Furthermore, we observed showed that the expression of *SMK2* in *smk2* heterozygous was dramatically decreased compared with that of W22 under salt stress or MV treatment (Fig. S6). We further detected the content of VB_6_ by using HPLC, and we observed that both NaCl and MV significantly increase VB_6_ (PL, PN, PM and PLP) content in W22 plants, but failed to do so in *smk2* heterozygous (Fig. 4D), which indicated that *SMK2* positively regulates VB_6_ accumulation and maize root growth under salt stress.

**Fig. 5:**
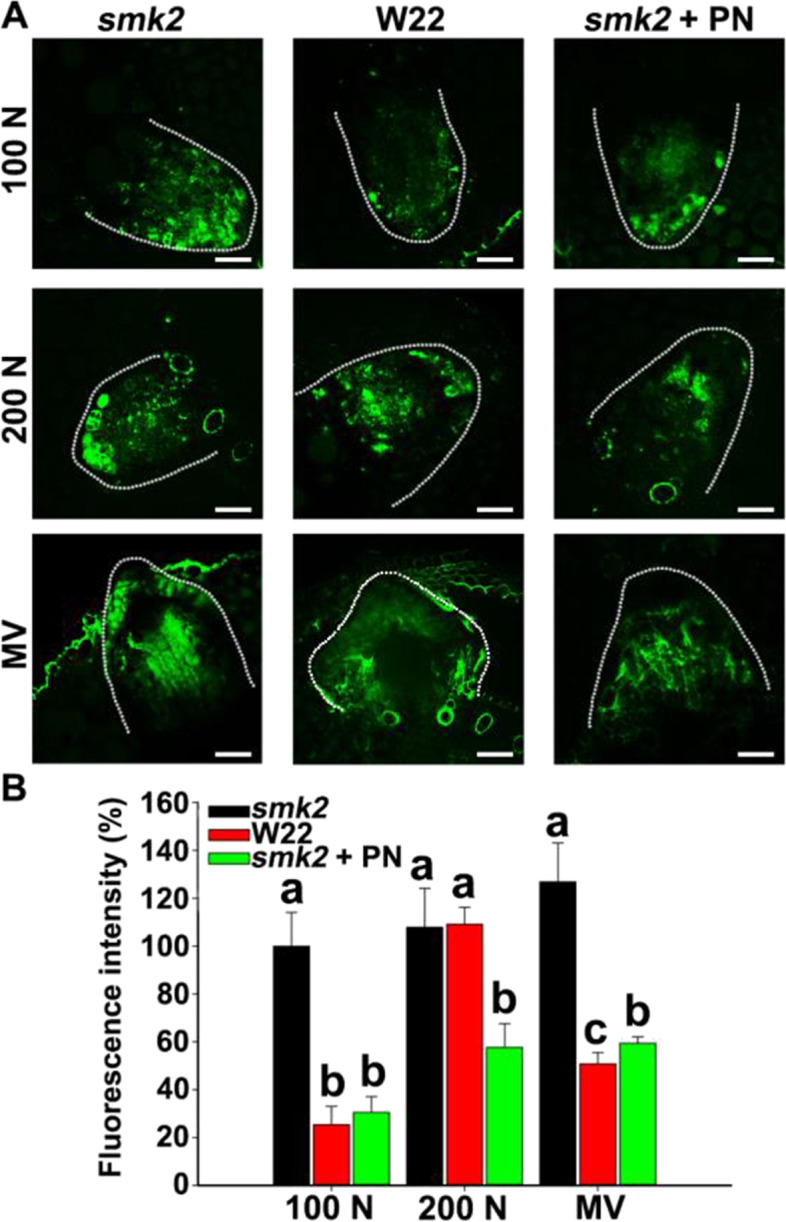
PN is extremely essential for *smk2* heterozygous plants to scavenge excessive ROS induced by NaCl and MV. **A** ROS fluorescence in LRs of 6-d-old *smk2* heterozygous and W22 seedlings subjected to 100 mM NaCl (N), 200 mM NaCl, 10 μM MV (methyl viologen) or added with 100 μM PN treatments for 24 h by using the fluorescent dye H_2_DCFDA. Bar = 50 μm. The white thread represents the profile of the LR. **B** ROS fluorescence intensity of (A). Image J software was used for quantifying fluorescence intensity. The different letters represent significant differences (*P* < 0.05, based on one-way ANOVA)

Furthermore, we detected the accumulation of ROS in W22 and *smk2* heterozygotes in the presence of NaCl and MV. We observed that NaCl and MV induced more ROS accumulation in *smk2* than that of W22 (Fig. 5A and B). In addition, the application of exogenous PN reduced ROS accumulation to a level similar to that of W22 (Fig. 5A and B), suggesting that PN is essential for plants to scavenge excessive ROS induced by salt stress. However, PLP failed to scavenging excessive ROS or restoring ABA biosynthesis genes (*AAO3, ZEP* and *VP14*) expression under salt/oxidative stress (Figs. S2 and S7), which suggest that PLP plays a different role than PN.

### PLP is essential for abscisic aldehyde oxidase activity

**Fig. 6:**
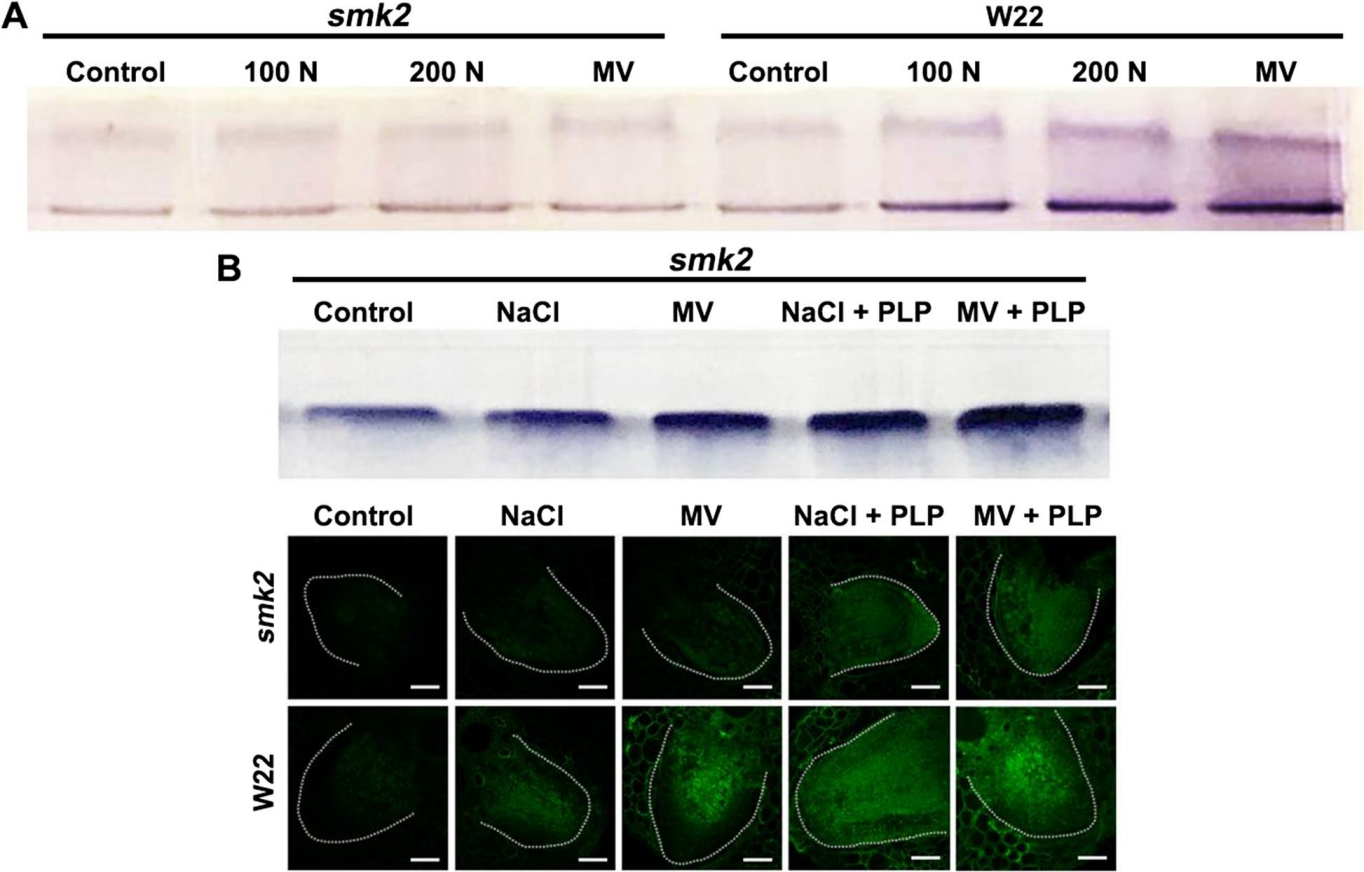
PLP was essential for AAO activity. **A** AAO activity of *smk2* heterozygous and W22. 6-d-old maize seedlings were treated with 100 mM NaCl (N), 200 mM NaCl and 10 μM MV (methyl viologen) for 24 h. The maize seedlings roots were harvested for detection of AAO activity. **B** AAO activity of *smk2* heterozygous. 6-d-old maize seedlings were treated with H_2_O (Control), 200 mM NaCl (N), 10 μM MV (methyl viologen), 200 mM NaCl + 100 μM PLP and 10 μM MV + 100 μM PLP for 24 h. The maize seedlings roots were harvested for detection of AAO activity. **C** ABA detection in *smk2* heterozygous and W22 maize LRs. 6-d-old *smk2* heterozygotes and W22 subjected to H_2_O (Control), 200 mM NaCl (N), 10 μM MV (Methyl viologen), 200 mM NaCl + 100 μM PLP and 10 μM MV + 100 μM PLP for 24 h. ABA fluorescence was detected by immunofluorescence in LRs. Bar = 50 μm. The white thread represents the profile of the LR

Previous results showed that PLP serves as an ABA3 cofactor, which encodes molybdenum cofactor sulfurase (Mendel and Bittner, 2006). In *A. thaliana*, ABA3 directly promotes the activity of aldehyde oxidase (AAO) (Bittner et al., 2001), which is involved in the conversion of ABA-aldehyde to ABA (Vishwakarma et al., 2017). As expected, we found that AAO activity in W22 was much higher than that of *smk2* heterozygous after NaCl or MV treatment (Fig. 6A). Furthermore, exogenous PLP partially recovered AAO activity under MV and NaCl treatment in *smk2* (Fig. 6B). Similarly, MV and NaCl failed to induce ABA accumulation in *smk2* roots, and exogenous PLP also restored the accumulation of ABA in *smk2* heterozygous LRs (Fig. 6C). Moreover, previous results showed that the content of PLP was increased 1.75-, 2.6- and 3.5-fold by NaCl (100 mM, 200 mM) and MV in W22 but was disabled in *smk2* heterozygous plants (Fig. 4D). These results indicate that PLP is essential for AAO activity, which further promotes ABA accumulation under oxidative stress.

## Discussion

Previous studies showed that VB_6_ biosynthesis gene was involved in plant defense against abiotic stress. VB_6_ biosynthesis-deficient mutant *pdx1.3* is sensitive to salt stress in Arabidopsis (Titiz et al., 2006), and overexpression of *PDX* genes enhanced plant resistance to salt stress (Bagri et al., 2018; Raschke et al., 2011), which is consistent with our research results. In addition, *PDX1.1* confers plant resistance to ammonium-induced oxidative stress by mediating VB_6_ biosynthesis (Liu et al., 2022b). However, whether the involvement of VB_6_ in the salt stress response requires the participation of ROS and ABA has not been reported. In summary, our study reveals that the important role of PN and PLP in balancing the dynamic equilibrium of ROS and ABA under salt/oxidative stress in maize.

### ROS acts as a bridge in salt stress-induced ABA and VB6 accumulation

Previous research indicated that both ROS and ABA were also induced by salt stress (Hao et al., 2021), and our study also indicated that VB_6_ accumulated under salt stress (Fig. 2B). ABA biosynthesis genes, such as *ZEP* and *VP14,* and ABA contents, were significantly induced by salt/oxidative stress (Fig. 1A and F). ROS scavenger CAT obviously decreased the expression of ABA biosynthesis and ABA contents (Fig. 1A and F). These results indicate that salt stress induces ABA accumulation by enhancing the expression of ABA biosynthesis genes, and this process requires excessive ROS accumulation. In addition, we also found that salt/oxidative stress upregulated the expression of VB_6_ biosynthesis genes *SMK2* and increased VB_6_ content, which was inhibited by CAT (Fig. 2A and B). Accordingly, ROS act as a positive inducing signaling molecule involved in salt-induced VB6 and ABA accumulation under salt stress.

### PN scavenges excess ROS accumulation to alleviates the inhibition of root development by salt stress

ROS act as a secondary messenger to regulating plant growth and development under various stresses (Bailey-Serres and Mittler, 2006). There are two ROS scavenging mechanisms in plants: an enzymatic scavenging system, including superoxide dismutase (SOD) and CAT (Gill and Tuteja, 2010), and a nonenzymatic scavenging system, mainly containing ascorbic acid, carotenoids, tocopherols, and flavonoids glutathione (You and Chan, 2015). Interestingly, our data showed that exogenous PN effectively decreased the production of excessive ROS induced by salt/oxidative stress (Figs. S2, Fig. 3A and D). Similarly, overexpression of the VB_6_ biosynthesis gene (*PDX-II*) enhanced potato tolerance to abiotic stresses (Bagri et al., 2018). In Arabidopsis, *pdx1.1* and *pdx1.3* are essential for root growth and development, and *pdx1.3* is more sensitive than Col-0 to salt stress (Titiz et al., 2006). Similarly, we found that salt stress induces high-level accumulation of PN, which was compromised in *smk2* heterozygous (Fig. 4D). In addition, *smk2* heterozygous roots were more sensitive to salt stress and oxidation stress, and supplementation with exogenous PN recovered the salt tolerance of *smk2* (Fig. 4A-C). Moreover, we also observed that *smk2* heterozygous accumulates more ROS than that of W22 under salt/oxidative stress, while exogenous application of PN can greatly reduce ROS accumulation (Fig. 5). Similarly, exogenous PN decreased salt/oxidative stress-induced the upregulation of ABA biosynthesis genes expression and ABA accumulation, which further relieved the inhibition phenotype of maize roots by salt/oxidative stress (Fig. 3, Figs. S3 and S4). Accordingly, we proposed that *SMK2* plays an essential role for plant defense against salt/oxidative stress, PN decrease the accumulation of ABA by scavenging excessive ROS, which further regulate root development under salt stress.

### PLP plays a novel regulatory role in ABA biosynthesis

Our results showed that ROS accumulated to higher levels and ABA biosynthesis genes were also induced in *smk2* heterozygous under NaCl or MV treatment (Fig. 5 and Fig. S7), which indicates that VB_6_ act as an antioxidant to scavenging excessive ROS and further regulating the accumulation of ABA. However, PLP failed to scavenging excessive ROS, relieving the inhibitory effect of salt stress on roots or decreased the upregulation expression of *ZEP*, *AAO3* and *VP14* induced by salt stress (Figs. S2, S3 and S7), which indicate PLP play a novel role in plant-salt/oxidative stress interaction. PLP is an active form of PL involved in over 170 enzymatic reactions as a cofactor (Rosenberg, 2012). PLP was indeed identified to be the molybdenum cofactor sulfurase ABA3-bound chromophore by binding with a NifS-like domain (Heidenreich et al., 2005). ABA3 directly activates aldehyde oxidase (AO), which was proposed to be involved in the conversion of ABA-aldehyde to ABA in plants (Bittner et al., 2001). Here, we found that salt/oxidation stress increased the activity of AAO in the W22 but failed to induce AAO activity in *smk2* heterozygous (Fig. 6A). Moreover, exogenous PLP recovered the AAO activity induced by salt/oxidative stress in *smk2* heterozygous (Fig. 6A). Furthermore, salt/oxidative stress failed to inducing the accumulation of ABA in *smk2* heterozygous (Fig. 6C), which could be restored by exogenous PLP in maize roots (Fig. 6C). Our latest report suggests that VB_6_ regulates ABA biosynthesis to close stomata, thereby enhancing rice resistance to bacterial leaf streak (Liu et al., 2022a), which suggests that PLP may play a role in plant-pathogen interaction. Overall, PLP plays an essential role in plant-pathogen and plant-adversity interaction by directly promoting AAO activity to further regulating ABA biosynthesis.

### Model of VB_6_ modulation of plant antioxidant stress

**Fig. 7:**
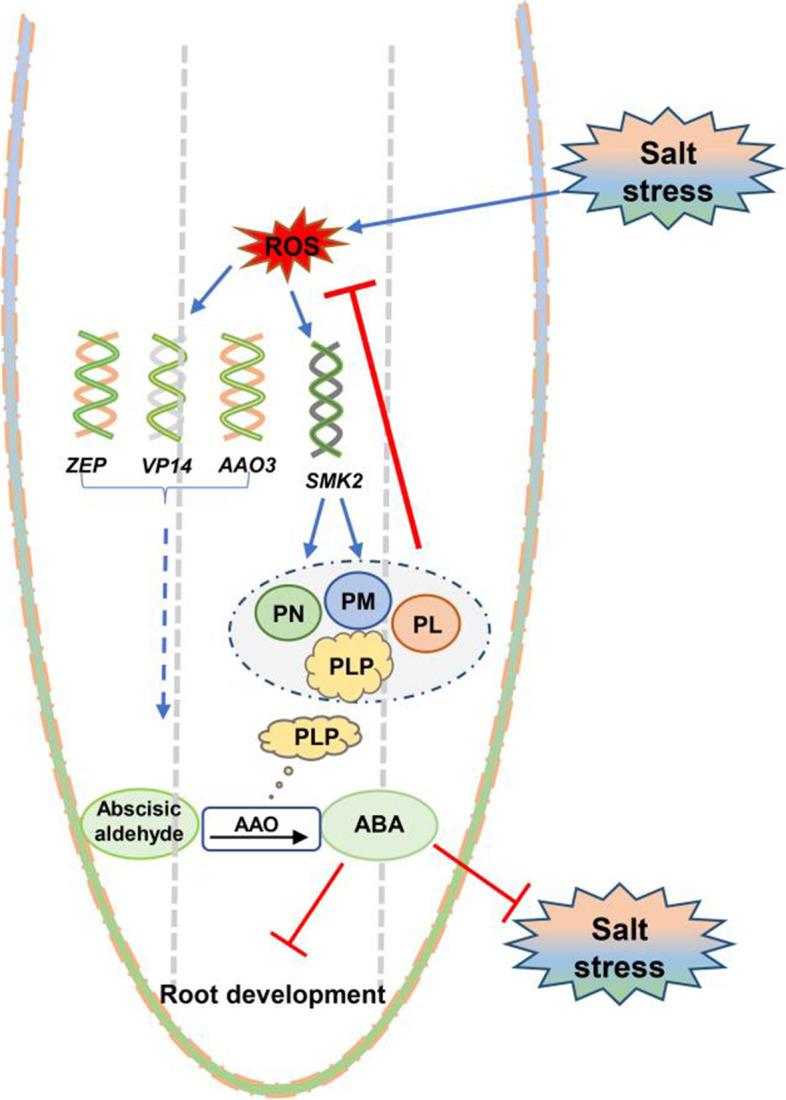
Possible model of VB6 involved in the plant response to salt stress. On the one hand, salt stress-induced ROS accumulation leads to oxidative damage and root inhibition, which further induces the expression of *ZEP*, *AAO3* and *VP14* and upregulates the VB_6_ (PL, PN, PM, PLP) content in maize roots. As a feedback mechanism, PN could scavenge excessive ROS to reduce the damage of oxidative stress to maize roots. On the other hand, PLP promoted AAO activity, further improving ABA production to inhibit root development and enhance plant salt stress tolerance

In conclusion, we generated the following possible model to better understand ROS-VB_6_-ABA interactions under salt/oxidative stress (Fig. 7). Salt/oxidative stress induces ROS accumulation, which next upregulates ABA- and VB_6_ biosynthesis-associated genes (*ZEP*, *VP14* and *SMK2*), and further increase VB_6_ (PL, PN, PM and PLP) content and ABA accumulation in maize roots. As feedback regulation, PN scavenges excessive ROS accumulation and alleviates its oxidative damage to maize roots. On the other hand, PLP acts as a coenzyme to activate AAO activity, which further promotes ABA biosynthesis. In a word, VB_6_ acts as a bridge to mediate the regulation of ROS on ABA accumulation under salt stress, so as to make maize roots better adapt to salt stress. Finally, our study revealed the role of VB_6_ in balancing the dynamic equilibrium of ROS and ABA under salt/oxidative stress.

## Materials and methods

### Plant growth condition

All maize seeds that were used in this study were surface-sterilized before use. The background of *smk2* heterozygous mutant used in this study is W22, and that of other maize seeding are B73 background, *smk2* homozygous mutants were embryo lethal, so we used *smk2* heterozygous mutants for the experiments (Yang et al., 2017). Specific operations were as follows: all maize seeds were surface-sterilized with 75% ethyl alcohol for 60 s, washed three times with deionized water, transferred to a 3% (v/v) NaClO solution for 10–15 min and washed 6–7 times in deionized water. All steps involved shaking and thorough mixing. Next, seeds were placed on a petri plate, which was covered by sterile absorbent cotton gauze, for germination and a proper amount of sterile double distilled water was added to keep the plate wet under a 16-h-light/8-h-dark photoperiod at 28 °C. Three days later, seedlings were transferred into Hoagland’s nutrient solution supplemented with different treatments.

### ROS detection

The ROS-specific fluorescence probe H_2_DCFDA (Invitrogen) was used to detect endogenous ROS. The maize seedling roots were washed with deionized water three times, and then the seedling roots were cut and sliced on clean glass slides. The transects of maize roots were placed into a 5 μM staining solution and was vacuumed for 15 min. Then, washed these samples for three times and imaged by a Zeiss LSM880 (Zeiss) confocal microscope (excitation, 488 nm; emission, 530 nm). For 3,3-diaminobenzidine (DAB) staining, the seedlings roots were put into 0.5 mg/ml DAB staining solution, vacuum for 0.5 hours (h). Then the samples were washed for three times with deionized water and reacted with H_2_O_2_ for 6–8 h under light. When the deep brown deposition appears in maize roots, transfer the samples into boiled ethanol and decolorizing three times. Finally, maize roots were imaged by a SMZ25 stereoscopic microscope (Nikon, Japan).

### Phenotypic analysis

Seedlings were transferred into 50%-strength Hoagland’s nutrient solution. The PR length was measured by a straightedge, and the number of LRs, which is emerged in PRs rather than adventitious roots, was counted with fine-tipped tweezers every day. The number of statistics was not less than 6 per experiment, and three parallel experiments were performed.

### Immunofluorescence assay of ABA

The immunofluorescence experiments used previous methods with some modifications (Ondzighi-Assoume et al., 2016). Maize seedling roots were washed three times to clean the roots. Then, roots were cut off from maize seedlings and placed on slides, and slicing was performed by hand to cut out the transverse section of the root. Next, the LR transverse sections were put into ABA stationary liquid and vacuum infiltrated for 2 h in an ice box. Then, the samples were transformed into a 4 °C refrigerator and left overnight, washed the samples for three times with 10 mM PBS (pH = 7.2) and then hyalinized in 10 mM PBS, including 0.2% pectinase, 0.2% cellulose, 3% (w/v) nonfat milk powder and 1% Triton X-100, for one hour at room temperature. The transverse sections were washed for three times and incubated with 1/3000 anti-ABA monoclonal antibody on a shaker at 4 °C overnight. Next, the samples were washed for many times to be cleaned and incubated with secondary antibody conjugated with Alexa Fluor 488 for 2 h at room temperature, and the formula of the incubation solution was the same except for the antibody. The transverse sections were washed 6–7 times to thoroughly remove antibody residues and blocked using Citifluor AF1 (Ted Pella, Inc.). Finally, pictures were imaged by using Zeiss LSM880 (excitation at 488 nm, emission at 510–560 nm). Fluorescence intensity was quantified using an analysis particle tool (ImageJ).

### Plant RNA extraction and quantitative PCR analysis

Total RNA of maize roots (~ 2000 ng) was extracted by using the TRIzon Reagent Kit (CWBIO). 1 μg total RNA was reverse transcribed for quantitative PCR by using a HiScript III 1st Strand cDNA Synthesis Kit (+gDNA wiper) Kit (Vazyme). Quantitative PCR analyses were conducted by using a SYBR Green Fast qPCR Mix (ABclonal). Target genes expression was calculated following the previous report (Chen et al., 2021).

### Vitamin B_6_ detection by HPLC

VB_6_ was extracted from maize roots. A 1200 HPLC series was used to quantitatively detect VB_6_ content. Three independent maize roots were obtained from three different experiments. VB_6_ was extracted by 1‰ (v/v) hydrochloric acid in darkness. The chromatographic column used by HPLC was a ZORBAXSB-C18 4.6*50 mm (Agilent Technologies, USA), the operating temperature was 25 °C, both mobile phases A and B were 1‰ (v/v) hydrochloric acid, the flow rate was 0.8 mL/min, the detection wavelength was 280 nm. Standard pyridoxal hydrochloride (CAS No: 65–22-5), pyridoxine hydrochloride (CAS No: 58–56-0), pyridoxamine dihydrochloride (CAS No: 524–36-7) and pyridoxal 5′-phosphate (CAS No:853645–22-4) were purchased from Sigma.

### Detection of AAO activity

AAO activity was detected refer to previously recent articles with minor modifications (Liu et al., 2022a). Maize roots were pretreated with NaCl, MV and other treatments and harvested, next, transfer the samples into liquid nitrogen and ground to a powder in a mortar, the powder was suspended in extraction buffer to extract total proteins, which were then subjected to native PAGE. The bands indicating AAO activity were developed with abscisic aldehyde (Sigma-Aldrich) in the reaction buffer (1 mM EDTA, 0.01 M flavin adenine dinucleotide, 2 mM dithiothreitol, 1 mM sodium molybdate, 0.05 M Tris HCl [pH = 7.5]) at 25–30 °C in the dark for 0.5 h.

## Supplementary Information

**Additional file 1: Table S1.** Primers for this article. **Fig. S1.** MV inhibit maize root development. (A) The phenotype of maize root subjected to 0, 0.1, 1 and 10 μM MV. (B) Number of the lateral roots (LRs) of (A). LRs is from independent maize seedling (*n* ≥ 6). The different letters represent significant differences (*P* < 0.05, based on one-way ANOVA). (C) The length of the primary roots (PRs) of (A). PRs is from independent maize seedling (n ≥ 6). **Fig. S2.** ROS is detected using DAB staining in maize roots. Seven-day-old maize roots subjected to 0, 200 mM NaCl (N), 200 mM NaCl supplemented with 100 μM PN, PM, PL and PLP or 10 μM MV, 10 μM MV supplemented with 100 μM PN, PM, PL and PLP for 24 h. DAB staining is used for ROS staining. **Fig. S3.** Exogenous PN enhance maize roots resistance to salt stress. (A) The phenotype of maize roots subjected to 200 mM NaCl, 200 mM NaCl + 100 μM PN, 200 mM NaCl + 100 μM PM, 200 mM NaCl + 100 μM PL and 200 mM NaCl + 100 μM PLP for 4 d. (B) PRs length of (A). PRs is from independent maize seedling (n ≥ 6). (C) Number of lateral roots of (A). LRs is from independent maize seedling (n ≥ 6). The different letters represent significant differences (*P* < 0.05, based on one-way ANOVA). **Fig. S4.  **The relative expression of *ZEP*, *AAO3* and *VP14.* Maize roots are subjected to H_2_O (Control), 100 mM NaCl (N), 200 mM NaCl + 100 μM PN, 10 μM Methyl viologen (MV) and 10 μM MV + 100 μM PN for 24 h, qRT-PCR is used to detecting the target genes expression. **Fig. S5.** PCR genotyping of *smk2* heterozygosity. Number 1–12 represent the different strains of maize. Number 1, 2, 3, 4, 6, 9, 10, 11 and 12 are identified as *smk2* heterozygosity. **Fig. S6.** The relative expression of *SMK2.* Maize roots are subjected to H_2_O (Control), 100 mM NaCl (N), 200 mM NaCl and 10 μM MV (Methyl viologen). qRT-PCR is used to detecting the target genes expression. **Fig. S7.** The relative expression of *ZEP*, *AAO3* and *VP14.* Maize roots are subjected to H_2_O (Control), 100 mM NaCl (N), 200 mM NaCl + 100 μM PLP, 10 μM Methyl viologen (MV) and 10 μM MV + 100 μM PLP for 24 h, qRT-PCR is used to detecting the target genes expression.

## Data Availability

All data and materials are available in the paper and online supplemental files.

## Abbreviations

ABA: Abscisic acid
ROS: Reactive oxygen species
VB_6_: Vitamin B_6_
PM: Pyridoxamine
PL: Pyridoxal
PN: Pyridoxine
PLP: Pyridoxal 5′-phosphate
SMK2: Small kernel2
LR: Lateral root
PR: Primary root
MV: Methyl viologen
HPLC: High-performance liquid chromatography
AAO: ABA-aldehyde oxidase
ZEP: Zeaxanthin oxidase
VP14: Viviparous14
PDX: Pyridoxine biosynthesis gene
DAB: 3,3-diaminobenzidine
MCSU: Molybdenum cofactor sulfurase
NCED: 9-cis-epoxycarotenoid dioxygenase
SOD: superoxide dismutase
CAT: Catalase
