# Supplementary material for: Multiple forms of vitamin B6 regulate salt tolerance by balancing ROS and abscisic acid levels in maize root
Source: Stress Biol. 2022 Sep 19;2(1):39. doi: 10.1007/s44154-022-00061-2 (PMC10441934; doi:10.1007/s44154-022-00061-2)
Supplement: Supplementary file 1 — Additional file 1: Table S1. Primers for this article. Fig. S1. MV inhibit maize root development. (A) The phenotype of maize root subjected to 0, 0.1, 1 and 10 μM MV. (B) Number of the lateral roots (LRs) of (A). LRs is from independent maize seedling (n ≥ 6). The different letters represent significant differences (P < 0.05, based on one-way ANOVA). (C) The length of the primary roots (PRs) of (A). PRs is from independent maize seedling (n ≥ 6). Fig. S2. ROS is detected using DAB staining in maize roots. Seven-day-old maize roots subjected to 0, 200 mM NaCl (N), 200 mM NaCl supplemented with 100 μM PN, PM, PL and PLP or 10 μM MV, 10 μM MV supplemented with 100 μM PN, PM, PL and PLP for 24 h. DAB staining is used for ROS staining. Fig. S3. Exogenous PN enhance maize roots resistance to salt stress. (A) The phenotype of maize roots subjected to 200 mM NaCl, 200 mM NaCl + 100 μM PN, 200 mM NaCl + 100 μM PM, 200 mM NaCl + 100 μM PL and 200 mM NaCl + 100 μM PLP for 4 d. (B) PRs length of (A). PRs is from independent maize seedling (n ≥ 6). (C) Number of lateral roots of (A). LRs is from independent maize seedling (n ≥ 6). The different letters represent significant differences (P < 0.05, based on one-way ANOVA). Fig. S4. The relative expression of ZEP, AAO3 and VP14. Maize roots are subjected to H2O (Control), 100 mM NaCl (N), 200 mM NaCl + 100 μM PN, 10 μM Methyl viologen (MV) and 10 μM MV + 100 μM PN for 24 h, qRT-PCR is used to detecting the target genes expression. Fig. S5. PCR genotyping of smk2 heterozygosity. Number 1–12 represent the different strains of maize. Number 1, 2, 3, 4, 6, 9, 10, 11 and 12 are identified as smk2 heterozygosity. Fig. S6. The relative expression of SMK2. Maize roots are subjected to H2O (Control), 100 mM NaCl (N), 200 mM NaCl and 10 μM MV (Methyl viologen). qRT-PCR is used to detecting the target genes expression. Fig. S7. The relative expression of ZEP, AAO3 and VP14. Maize roots are subjected to H2O (Control), 100 mM NaCl (N), 200 mM [file 44154_2022_61_MOESM1_ESM.docx]

**Table. S1 Primers for this article**

| Primers for qRT-PCR |  |
| --- | --- |
| SMK2-qF | GGAGAAGGAAGGAGGGAATG |
| SMK2-qR | GGGTGGAAAGCAGTTGCGAGGA |
| VP14F | TTTCTCCCTTTGGATGACAT |
| VP14R | CAAGTAACAGCAACCAAGAT |
| ZEPF | TCCCTATCATTGTCCTCTCCA |
| ZEPR | CTACCTTCATTGTCGGTAATC |
| AAO3-qF | CCTTGGCATTCCATTTCACA |
| AAO3-qR | GCAACATTGGGCTCACATCC |
| Actin1-qF | GATTCCTGGGATTGCCGAT |
| Actin1-qR | TCTGCTGCTGAAAAGTGCTGAG |
| For *smk2* heterozygous detection |  |
| SMK2-RTF1 | GCTTCTTTCTCCCCTTCCAT |
| SMK2-RTR1 | GATTGTGGTAGTTGGCGAGCTT |
| TIR8.1 | CGCCTCCATTTCGTCGAATCCCCTS |


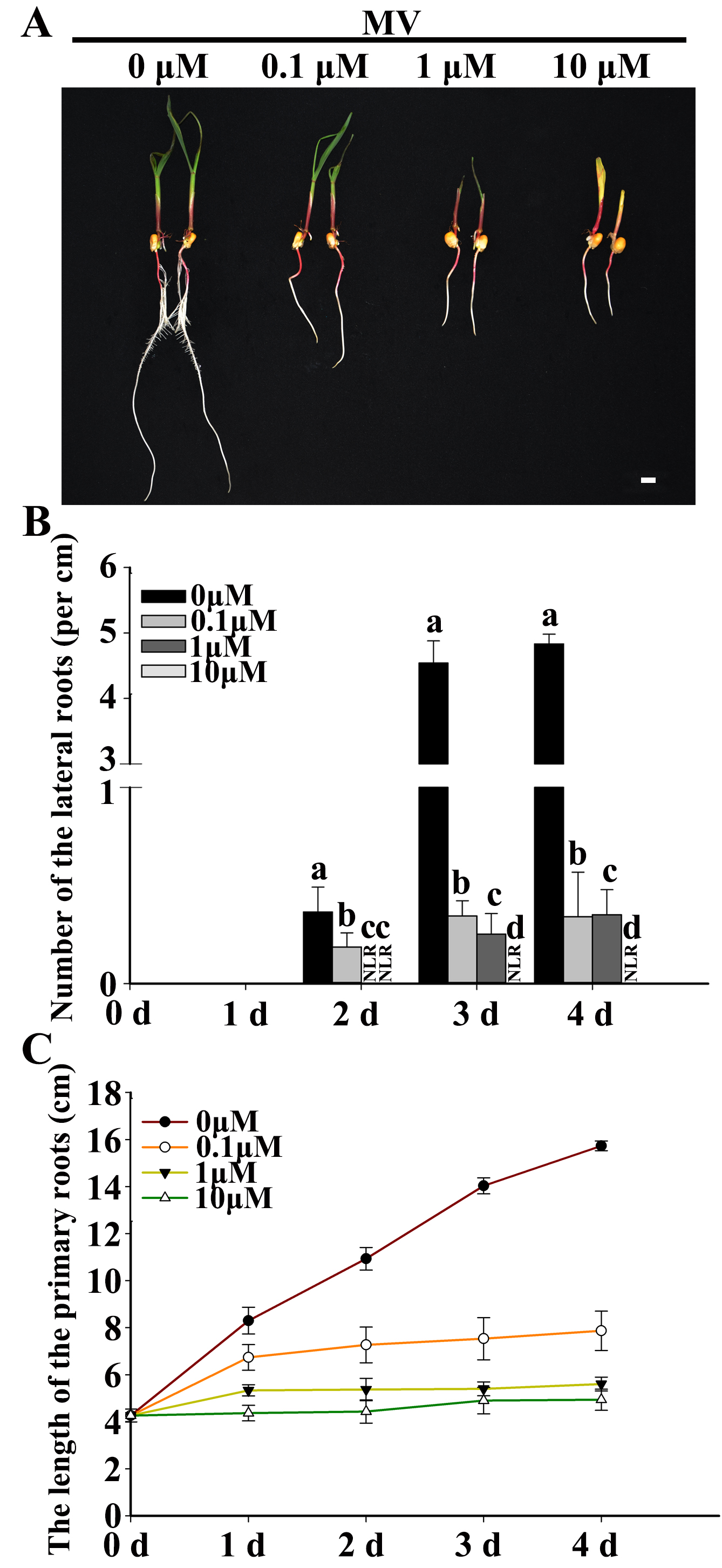


**Fig. S1 MV inhibit maize root development**

1. The phenotype of maize root subjected to 0, 0.1, 1 and 10 μM MV.
2. Number of the lateral roots (LRs) of (A). LRs is from independent maize seedling (n ≥ 6). The different letters represent significant differences (*P* < 0.05, based on one-way ANOVA).
3. The length of the primary roots (PRs) of (A). PRs is from independent maize seedling (n ≥ 6).

**
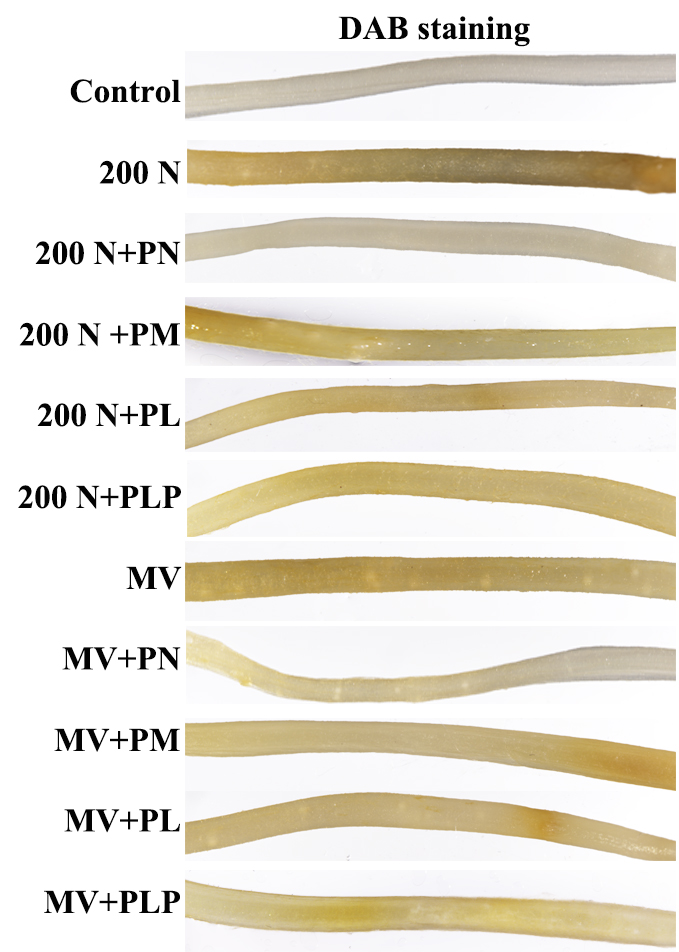
**

**Fig. S2 ROS is detected using DAB staining in maize roots**

Seven-day-old maize roots subjected to 0, 200 mM NaCl (N), 200 mM NaCl supplemented with 100 μM PN, PM, PL and PLP or 10 μM MV, 10 μM MV supplemented with 100 μM PN, PM, PL and PLP for 24 h. DAB staining is used for ROS staining.


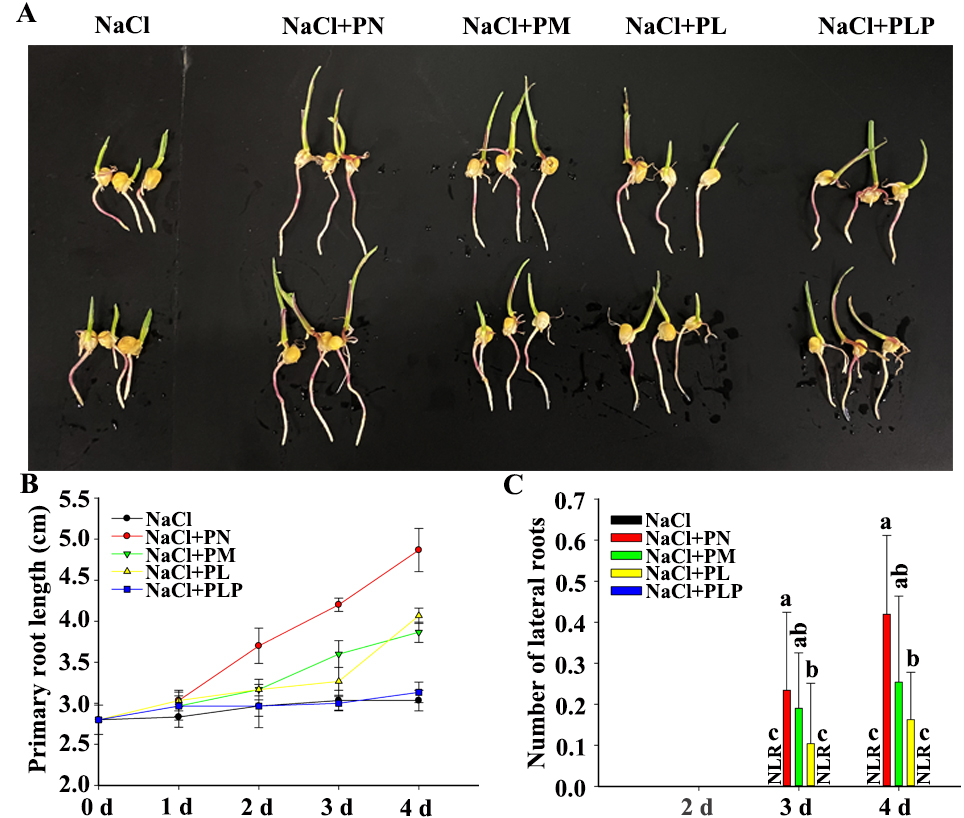


**Fig. S3** **Exogenous PN enhance maize roots resistance to salt stress.**

1. The phenotype of maize roots subjected to 200 mM NaCl, 200 mM NaCl + 100 μM PN, 200 mM NaCl + 100 μM PM, 200 mM NaCl + 100 μM PL and 200 mM NaCl + 100 μM PLP for 4 d.
2. PRs length of (A). PRs is from independent maize seedling (n ≥ 6).
3. Number of lateral roots of (A). LRs is from independent maize seedling (n ≥ 6). The different letters represent significant differences (*P* < 0.05, based on one-way ANOVA).


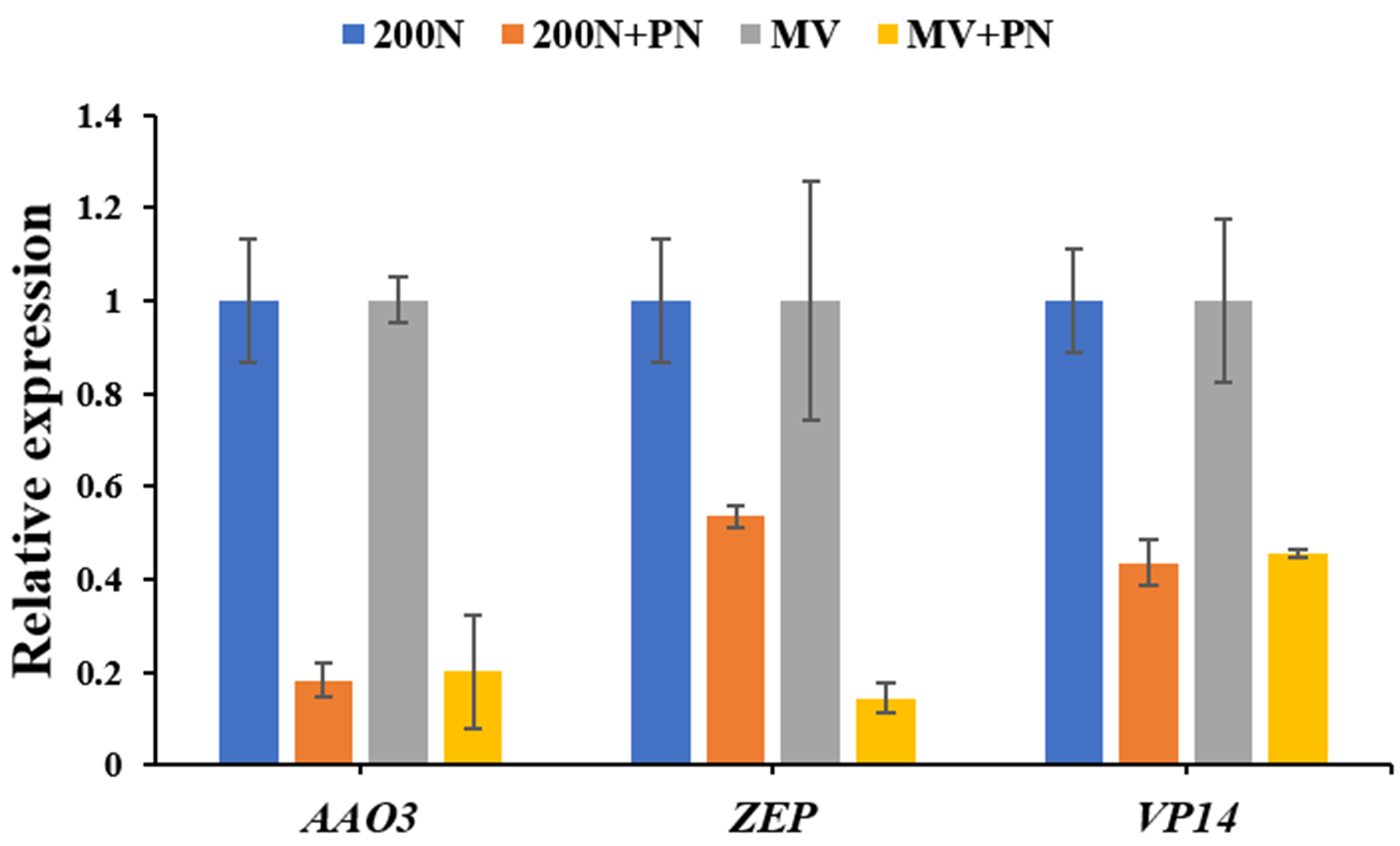


**Fig. S4 The relative expression of *ZEP*, *AAO3* and *VP14* in maize roots subjected to H_2_O (Control), 100 mM NaCl (N), 200 mM NaCl + 100 μM PN, 10 μM Methyl viologen (MV) and 10 μM MV + 100 μM PN for 24 h.**


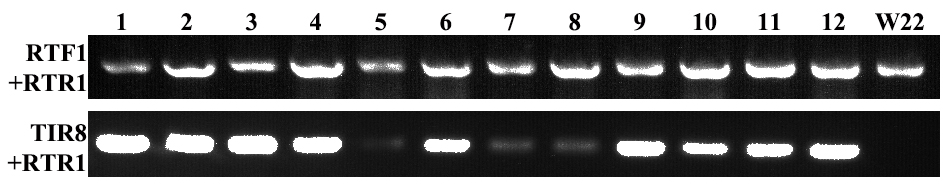


**Fig. S5 PCR genotyping of *smk2* heterozygosity.**

Number 1-12 represent the different strains of maize. Number 1, 2, 3, 4, 6, 9, 10, 11 and 12 are identified as *smk2* heterozygosity.


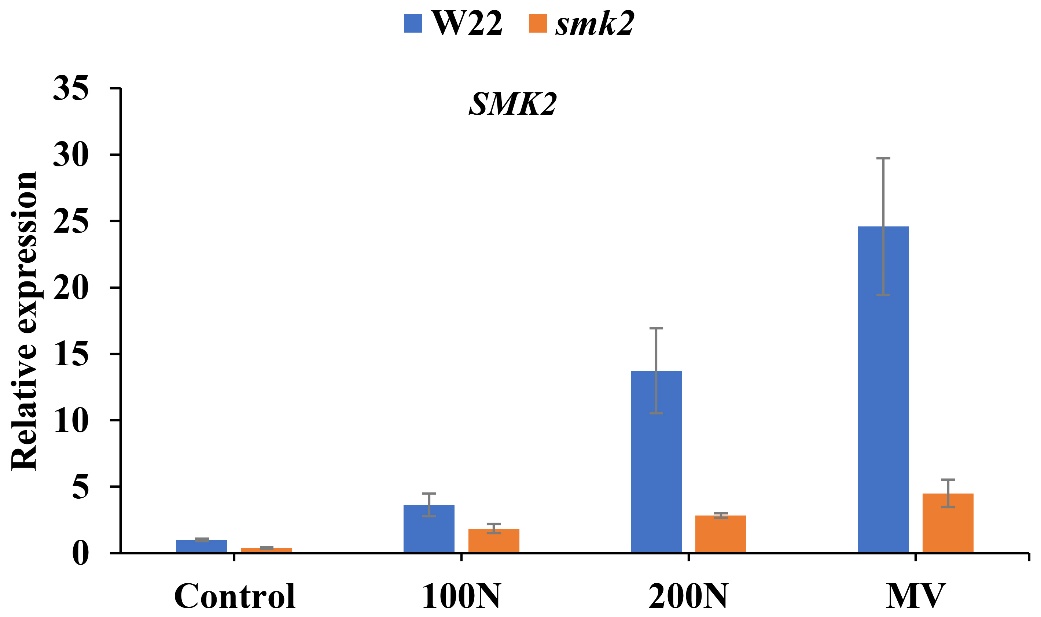


**Fig. S6 The relative expression of *SMK2* in maize roots subjected to H_2_O (Control), 100 mM NaCl (N), 200 mM NaCl and 10 μM MV (Methyl viologen).**


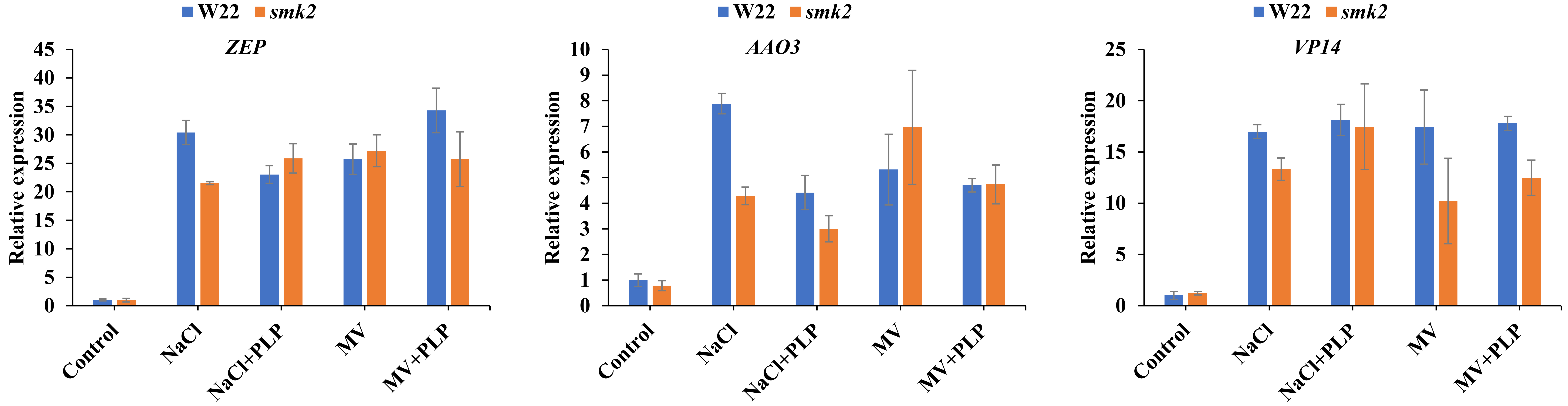


**Fig. S7 The relative expression of *ZEP*, *AAO3* and *VP14* in maize roots subjected to H_2_O (Control), 100 mM NaCl (N), 200 mM NaCl + 100 μM PLP, 10 μM Methyl viologen (MV) and 10 μM MV + 100 μM PLP for 24 h.**
